# Kinematics and Mechanical Properties of Knees following Patellar Replacing and Patellar Retaining Total Knee Arthroplasty

**DOI:** 10.1155/2015/391450

**Published:** 2015-11-30

**Authors:** Rongying Huang, Yanqiang Liu, Jun Zhu

**Affiliations:** School of Mechanical Engineering and Automation, Beihang University, Beijing 100191, China

## Abstract

Knee injury is a common medical issue. A full understanding of the kinematics and mechanical properties of knees following total knee arthroplasty (TKA) repair utilizing patellar replacement (only the base of the patella is replaced) versus patellar retaining surgical techniques is still lacking. In the current paper, we investigated magnetic resonance (MR) imaging data from knees repaired by these two methods and evaluated total knee models created using imaging reconstruction technology that simulated gait conditions. Results revealed that patellar replacement had little influence on tibiofemoral kinematics, although the tibia-surface equivalent stress increased slightly. By contrast, patellar replacement had a significant influence on the patellofemoral joint; patellar internal rotation, external rotation, and medial-lateral translation were all increased. Moreover, the stress distribution on patellar prostheses was altered, resulting in an increased surface maximal equivalent stress on the corresponding area. Moreover, during the gait cycle, we found that the area with maximal equivalent stress shifted its position. Finally, the patellofemoral joint showed decreased motion stability. From the view of kinematics and mechanics, this paper suggests that patella should be retained during TKA if it is possible. The present study presented approaches and technologies for evaluating kinematics and mechanical properties of total knee joint after TKA under gait loads.

## 1. Introduction

As a major supporting joint of human movement, the knee is prone to damage. Ultimately, the best treatment method for repairing knee articular surface damage is total knee arthroplasty (TKA), which replaces the articular surface with knee prosthesis, thereby restoring knee joint functions. With the constant improvement in knee surgery techniques and the demanding requirements of joint prostheses to accurately mimic the knee function, the problems concerning knee kinematics under gait dynamic loading and contact properties following TKA have come under close scrutiny.

For instance, in 2002, Godest et al. [1] first introduced the finite element method and studied the mechanical properties of femoral and tibial implants under gait loads in TKA knees. Subsequently, in 2005, Halloran et al. [2] conducted finite element analyses of knees following TKA, the results of which closely predicted the results from a concurrent experimental study. In 2007, Knight et al. [3] extended the application of finite element method to biomechanical issues by investigating implant wear following TKA. As such, in 2012, Wachowski et al. [4] examined the effects of knee prostheses with roll back characteristics on patellofemoral joint forces and showed that the prosthesis alleviated joint pain in TKA patients and elongated the service life of the prosthetic. On a similar note, in 2011, Walker et al. [5] established criteria for evaluating knee prosthesis through studying the kinematics of three different knee prostheses.

The knee joint is a very complex structure. The complicated coupling and coordinating relationship between the tibiofemoral and patellofemoral joints during movement/gait makes it difficult to study the kinematics and mechanical characteristics of TKA-repaired knees under dynamic gait loading conditions. In this study, we analyzed and compared the total knee joint kinematics and mechanical properties during gait cycle following patellar replacement versus patellar retaining TKA. Then the influence of patellar replacement and patellar retaining TKA on total knee functions was assessed. The research steps include knee joint modeling, mechanical simulation of patellar retaining or replacement PS-type prostheses under dynamic gait loads, and the comparison of tibiofemoral and patellofemoral kinematics and contact stresses of knees with PS-type prostheses. The results of this study will provide novel techniques and methods for evaluating the effects of replacement surgery on knee kinematics and mechanical properties.

## 2. Methods

### 2.1. TKA Knee Joint Modeling

The knee joint model contained two parts.

(*1) PS Prosthesis Modeling*. The PS prosthesis was provided by Beijing Chunlizhengda Medical Apparatus Inc. A MicroScribe G2 three-dimensional scanner was employed to scan every structure of the total knee prosthesis to obtain geometry, which was then imported into Geomagic Studio 8.0 for further optimization and also into Solidworks for model repair simplification and reconstruction.

(*2) Knee Modeling after TKA*. Following TKA, the knee model consisted of either a patellar retaining or PS prosthesis replacing knee joint model. Initially, a normal knee joint model was created based on CT and MR data of the normal knee. Subsequently, the femur and tibia were cut according to the surgery procedure [6]. Finally, the remaining femur, tibia, and PS prostheses were assembled to create the total knee model. The normal knee joint MR data was acquired by scanning a student volunteer under the supervision of a doctor.

### 2.2. Total Knee Model after TKA

The simulation of the total knee joint after TKA consisted primarily of model simplification, finite element modeling, and definition of dynamic gait loading.

(*1) Knee Model Simplification*. According to [7–11], the ligaments (anterior cruciate (ACL), posterior cruciate (PCL), medial collateral (MCL), and lateral collateral (LCL)) and surrounding muscles were represented by springs; specifically, the quadriceps femoris and patellar ligament were represented by 2 bundles of spring elements with stiffness coefficients of 2000 N/mm and 1142 N/mm, respectively. The MCL was represented by three bundles of spring elements (i.e., anterior bundle, deep bundle, and oblique bundle), while the LCL was comprised of a single bundle of nonlinear spring elements. The medial/lateral springs' stiffness coefficients and deformations were from [9–11] and shown in Table 1.

(*2) Total Knee Finite Element Model*. The finite element model was created by using ABAQUS 6.11 [5, 10–14], including the definition of material properties, connection relation, boundary conditions and constraints, and grid generation.

The bone tissue is simplified as isotropic linear elastic material with the elastic modulus of 17 GPa and Poisson's ratio of 0.3. Femoral prosthesis is cobalt-molybdenum alloy with the elastic modulus of 79 GPa and Poisson's ratio of 0.3. The tibial tray prosthesis is titanium alloy with the elastic modulus of 70 GPa and Poisson's ratio of 0.3. The UHMWPE (ultra-high-molecular-weight polyethylene) tibial insert and the patellar prosthesis use nonlinear elastoplastic deformable material properties.

Different coupling relationship between the components of the knee prosthesis is defined. The relationships between the femoral prosthesis and the femoral condyle section, tibial prosthesis and tibial section, and tibial prosthesis and tibial liner are defined as consolidation. The contacts between the femoral prosthesis and polyethylene insert, patella cartilage, and femoral prosthesis were defined as sliding friction, with a friction coefficient of 0.004 [1, 2, 9].

The definition of the boundary conditions and constraints is as follows: the patella is not limited, the movement of femoral with prosthesis is limited to flexion and extension, the tibial with prosthesis is only limited with the outer flexion and extension, and turning and shift movement on the other directions are not limited.

**Figure 1:**
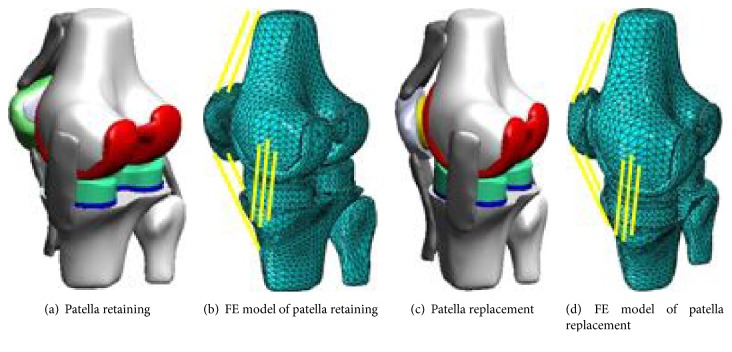
3D and FE model following TKA with prosthesis.

C3D10 tetrahedron grid elements were employed to achieve free mesh generation, and the models of patella retaining and replacement TKA were divided into 81052 and 78397 elements, respectively. The total knee after TKA and the corresponding finite element models are shown in Figure 1.

(*3) Applying Dynamic Loading*. There were three main processes utilized when the gait dynamic loads were applied.

**Figure 2:**
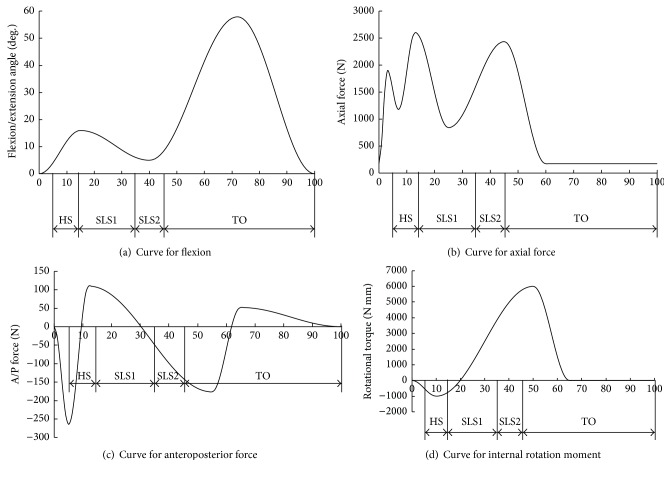
The distribution of gait loading curves during gait cycle.

(1) According to the standard ISO 14243-1, the gait cycles were divided into four main states [12, 13, 15–19]: heel strike (HS), single limb stance 1 (SLS1), single limb stance 2 (SLS2), and toe off (TO). The gait loading curve was also divided into four areas (as shown in Figure 2) to analyze the movement and mechanical characteristics of the human gait cycle after TKA.
(2) The loading curve was transferred into discrete data by using piecewise polynomial fitting techniques to actualize the human knee gait cycle motion simulation of dynamic loading after TKA. The gait flexion and gait load were controlled by fitting functions programmed with Matlab, including gait flexion function, axial force loading function, anteroposterior force loading function, and the internal and external rotation torque loading function.
(3) A gait dynamic load was applied to the total knee after TKA. The line connecting the rotational centers of the femoral lateral and medial epicondyles was defined as the axis for femur flexion (Figure 2(a)), and axis force was applied to the centers of each of the femoral lateral and medial epicondyles based on the axis force loading function (Figure 2(b)), where 60% axis force was applied to the center of the medial epicondyle and 40% axis force was applied to the lateral epicondyle [20]. The anteroposterior force (Figure 2(c)) and internal rotation moment (Figure 2(d)) were applied to the tibia implant based on the anteroposterior force function and internal-external rotation function. All the *x*-axes of Figures 2(a)–2(d) are percentage of gait cycle time.

### 2.3. Dynamic Simulation of the Total Knee with Patellar Retaining and Replacement under Gait Loading

The dynamic simulation of the total knee with patellar retaining and replacement during gait loading was performed in ABAQUS 6.11 by means of explicit dynamic finite element analyses. The data were collected at a 5% gait cycle interval. The effects of the patellar treatment method on joint function were analyzed by comparing the equivalent contact stress value on the articular surface and motion characteristics of the tibiofemoral joint and patellofemoral joint.

## 3. Results

### 3.1. Kinematics of Total Knee Joint after Patellar Retaining and Replacement TKA

#### 3.1.1. Kinematics of the Tibiofemoral Joint under Gait Loads

**Figure 3:**
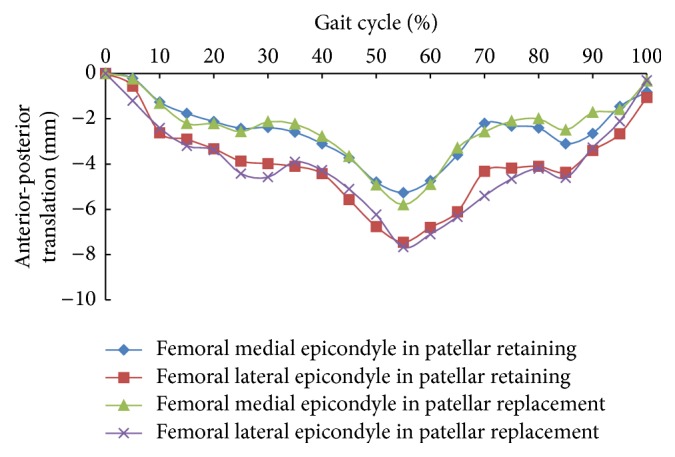
Anterior-posterior translation of femoral lateral and medial epicondyles during the gait cycle.

**Figure 4:**
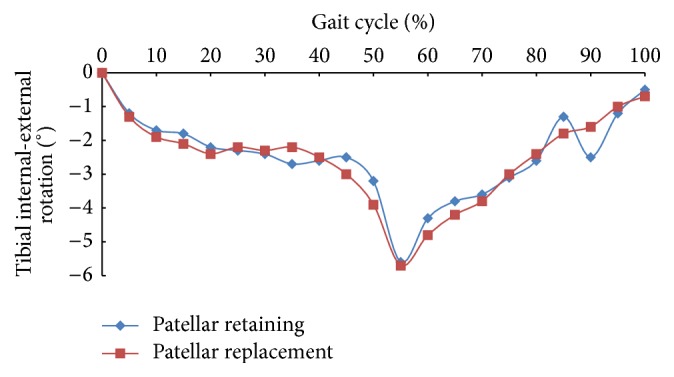
Tibial internal-external rotation for patellar retaining and replacement models.

The relative movement curves of the tibiofemoral joint from simulation results of both models are shown in Figures 3 and 4. Results revealed that both patellar retaining and patellar replacement had little influence on femoral anteroposterior translation. Similar value and tendency could be observed for the two models in terms of anteroposterior translation of the medial and lateral epicondyle center. During the entire gait cycle, both the lateral and medial femoral epicondyle translated posteriorly, peaking at about 55% of the gait cycle. For the patellar retaining model, the posterior translations of the femoral lateral and medial epicondyles were 5.3 mm and 7.5 mm, respectively, while in the patellar replacement model, the values were 5.8 mm and 7.7 mm, respectively. These results are similar to that found previously by Halloran et al. [2] who reported a maximal posterior translation of 5.1 mm at 60% of gait cycle.

For both models, both the tibial internal and external rotations and variations thereof showed similar results. At 0%–20% of the gait cycle, the tibia rotated internally by 2.2° and remained stable from 20% to 45% of the gait cycle, peaking at 55% (6°), at which point the internal rotation began to gradually decrease. These results are consistent with a previous report [1] that observed a maximal internal rotation of 5°.

The peak femoral posterior translation and tibial internal rotation both occurred at about 55% to 60% of the gait cycle, when the foot was lifted off of the ground and the tibial axis force was small. The body's center of gravity transferred, and the knee joint was subject to a moment of high internal rotation and posterior force.

#### 3.1.2. Kinematics of the Patellofemoral Joint under Gait Loads

**Figure 5:**
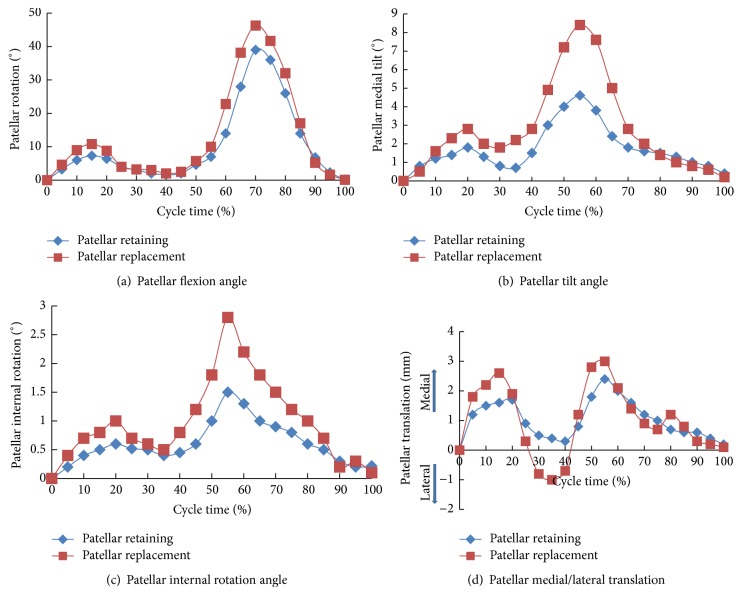
Kinematics of the patellofemoral joint.

Figure 5 shows the patellofemoral kinematics during the gait cycle for patellar retaining and replacement TKA knees. Patellar replacement TKA significantly increased patellar movements compared to patellar retaining TKA. Moreover, patellar tilting, internal rotation, and medial/lateral translation were all significantly higher. Patellar flexion was unchanged. Therefore, the patellofemoral joint motion stability was decreased following patellar replacement TKA. The patellar flexion variation curve revealed that the tibiofemoral joint flexion variation was slightly greater in the patellar replacement versus the patellar retaining model, with a maximal flexion angle of 39° and 46.3°, respectively, observed at 70% of the gait cycle.

For both models, the second highest and maximal patellar tilting occurred at 20% and 50% of gait cycle, respectively, which were equal to 1.8° and 4.6° for the patellar retaining model and 2.8° and 8.4° for the patellar replacement model. Patellar internal rotation showed a similar tendency as tilting; the second highest and maximal internal rotation at 20% and 55% of gait cycle were 0.6° and 1.5° for the patellar retaining model and 1° and 2.8° for the patellar replacement model.

The patellar lateral-medial translation showed evident differences between the two models, though the variation tendency was similar. The patellar first moved medially to the maximal value and then laterally till the minimal value was achieved before it moved medially to the maximal value once again. The peak, minimal, and maximal values occurred at 20% (1.7 mm), 35% (0.3 mm), and 55% (2.4 mm) of the gait cycle, respectively. For the patellar replacement model, the peak, minimal, and maximal values occurred at 15% (2.6 mm), 35% (1 mm), and 55% (3 mm) of the gait cycle, respectively, values significantly higher than those in the patellar retaining model indicating a decreased knee joint stability.

### 3.2. Mechanical Properties of TKA Knee Joint under Gait Loads

#### 3.2.1. Mechanical Properties of Tibiofemoral Joint under Gait Loads

The mechanical properties of the tibiofemoral joint under gait loads were investigated by analyzing the stress contour and maximal stress variations of the UHMWPE tibial tray during the gait cycle, thereby illustrating the mechanical differences between the tibiofemoral joints of two models.

**Figure 6:**
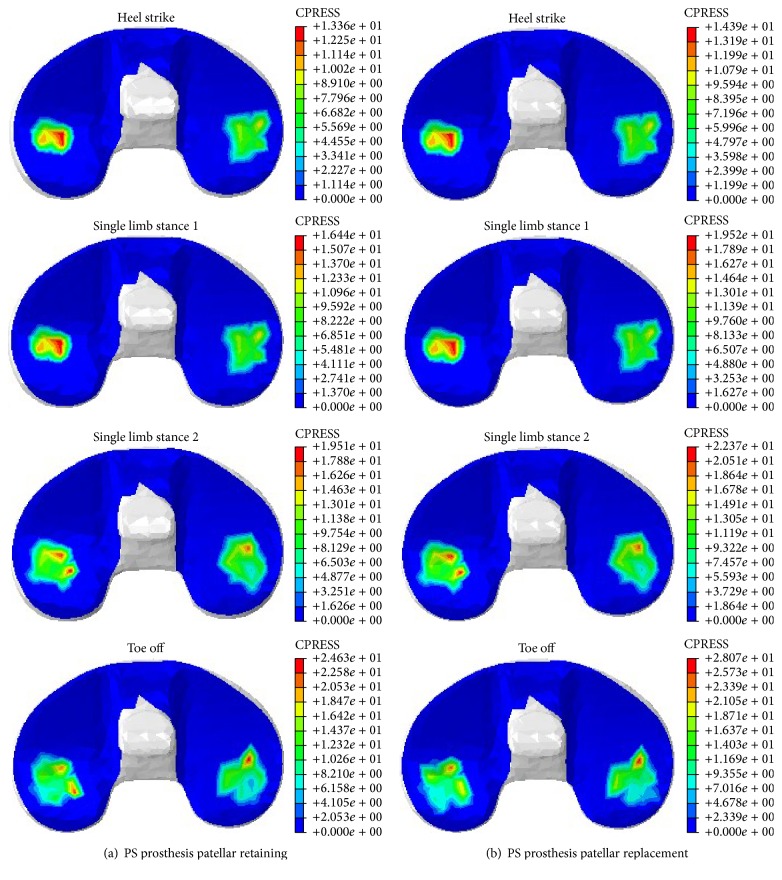
Stress contour of tibial tray during gait cycle.

The stress distributions of the UHMWPE tibial tray were represented by the stress contour at typical states, HS, SLS, and TO (Figure 6). Results revealed that the stress distributions of the two models are similar. During the gait cycle, high stress mainly concentrates on the tibial medial condyle (used dominantly during gait) at SLS2 and TO. In addition, the equivalent stress and equivalent stress area of the lateral condyle slightly increased probably due to the displacement of the center of gravity.

**Figure 7:**
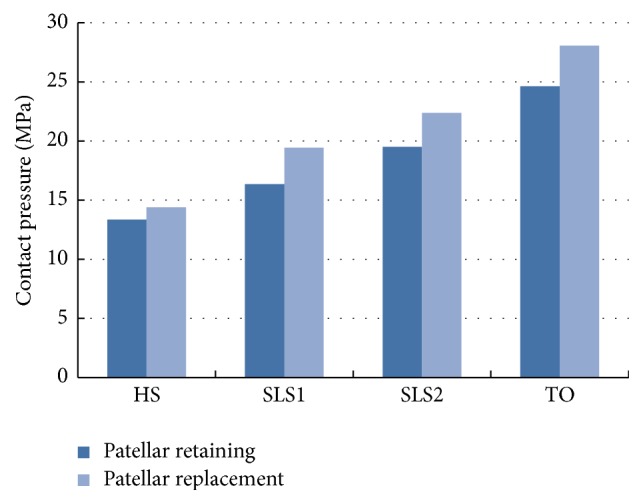
Histogram of the maximal contact stress of the UHMWPE tibial tray during the gait cycle.

A histogram of the maximal equivalent peak contact stress of the UHMWPE tibial tray during the gait cycle is shown in Figure 7. Combining Figures 6 and 7, the mechanical properties of the tibial tray are listed in Table 2. Table 2 suggested that although the stress distribution of tibial tray was similar for the two models, patellar replacement resulted in elevated stress, which may aggravate the tibial tray wear.

**Figure 8:**
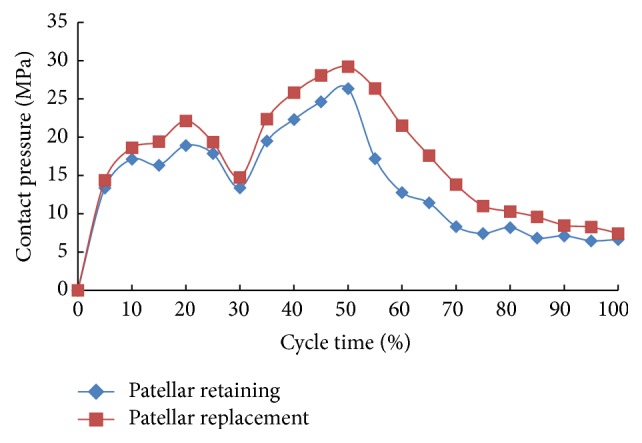
Variation in the peak contact stress of the tibial tray during the gait cycle for the patellar retaining versus patellar replacement TKA models.

Figure 8 shows the variation curve of tibial tray contact stress under gait loads. Results show that the two models demonstrated similar variation, peaking at 50% of the gait cycle and gaining a second maximal stress value at 20%. However, the stress was consistently higher in the patellar replacement model than in the patellar retaining model. For both models, during 0%–10% of the gait cycle, the heel strike imposed an increasing tibial axial force. Meanwhile, the tibial tray stress reached 17.1 MPa and 18.6 MPa for internal rotation movement and tibial anterior force, respectively. When the entire foot made contact with the ground the tibial axial force was slightly reduced due to forward body movement. As such, at 30% of the gait cycle the tibial tray contact stresses decreased correspondingly, with values of 13.4 MPa and 14.77 MPa for internal rotation movement and tibial anterior force, respectively. Then, as the foot began to push off of the ground and separation occurred, we found that the axial force increased, reaching maximal contact stress values of 26.35 MPa and 29.23 MPa for tibial movement and anterior force, respectively. After the foot lifted from the ground, the tibial axial force and tibial tray contact stress both decreased substantially. Taken together, these results reveal that the variation in the contact stress reflects the changes in the knee supporting force during gait.

#### 3.2.2. Mechanical Properties of Patellofemoral Joint after TKA

**Figure 9:**
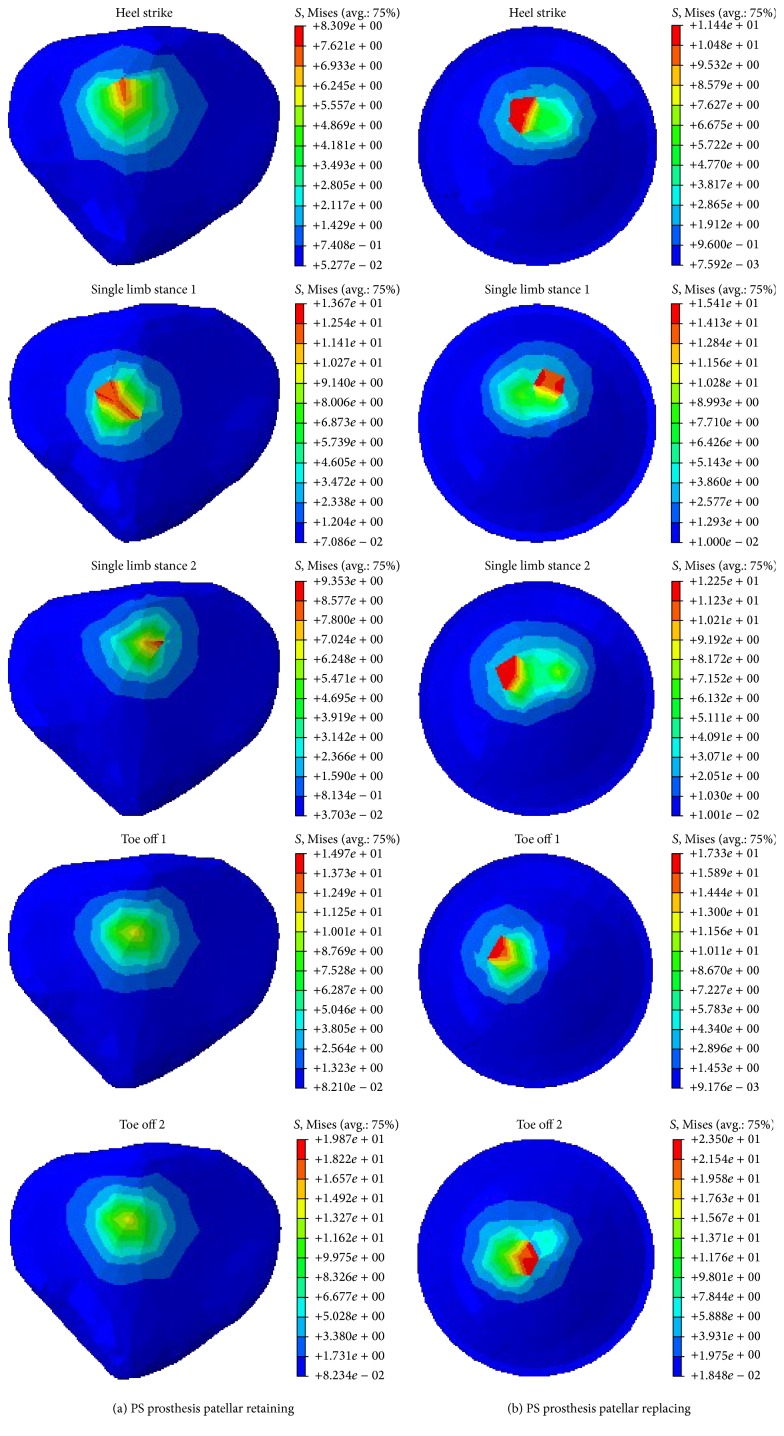
Contours of equivalent stress in patella and patellar implants during the gait cycle.

The mechanical properties of the patellofemoral joint were described by using a surface patellar stress contour, a histogram of maximal equivalent stress, and a maximal equivalent stress variation curve.  Figure 9 shows the stress contours of the patellar surface or patellar prosthesis surface. Considering the fact that the flexion angle can affect the patellar maximal equivalent stress, the toe off state was divided into two states: TO1 and TO2. At TO1, the tibiofemoral flexion angle was 8° and the axial force was 2434 N. Meanwhile, at TO2, when the foot is separated from the ground, the axial force reached 168 N with 56° flexion. It can be seen that compared to patellar retaining TKA, patellar replacement altered the stress distribution of the patellofemoral joint surface, resulting in an increased peak stress area, a maximal equivalent stress, and a significantly varying peak stress area.

**Figure 10:**
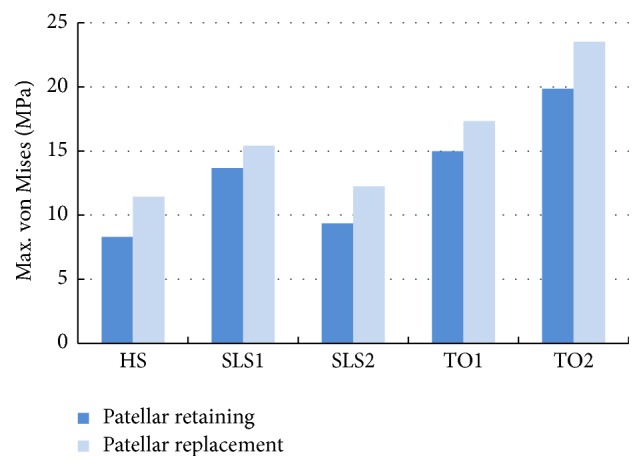
Bar diagram of peak equivalent stress of patella and patellar implant during gait cycle.

Combining Figures 9 and 10, the stress distribution of the patella and patellar implant was found to be related to the knee axial force and the tibiofemoral flexion angle in addition to the patellar treatment method. At higher axial force and tibiofemoral joint flexion, an increase in the peak equivalent stress was observed. Following patellar replacement TKA, the patellar implant showed significantly higher peak equivalent stresses, presumably due to changes in the contact surface and a loss of patellar cartilage for protective buffering. Specifically, at HS, the peak equivalent stress was 8.31 MPa and 11.44 MPa for the patellar retaining and patellar replacement model, respectively, an increase of 37.67%. At SLS1, the peak equivalent stress for the two models was 13.67 MPa and 15.41 MPa, respectively, an increase of 12.73%. At SLS2, the values were 9.35 MPa and 12.25 MPa, respectively, an increase of 31.02%. At TO1, the peak equivalent stress was 14.97 MPa and 17.33 MPa, respectively, an increase of 15.75%. Finally, at TO2, the peak equivalent stress was 19.87 MPa and 23.5 MPa, respectively, an increase of 18.27%. These results clearly reveal increased peak equivalent stresses in the patellar replacement model.

**Figure 11:**
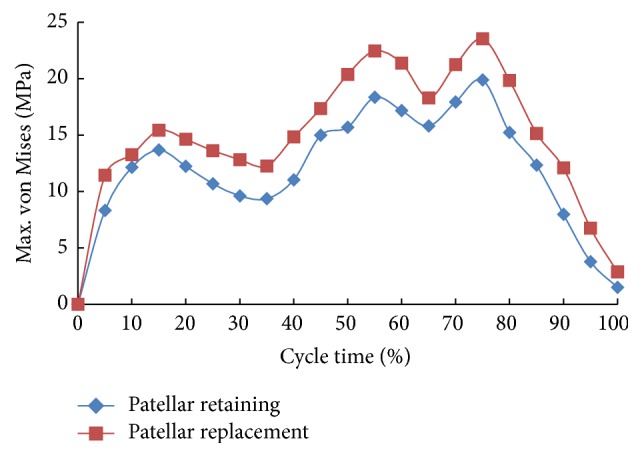
Peak equivalent stress curve of patella and patellar implant during gait cycle.

It can be seen from Figure 11 that the patella and patellar implant exhibited a similar variation in the peak equivalent stress during the gait cycle. However, the patellar implant stress was significantly higher. At 45% of the gait cycle the foot was separated from the ground, but due to the high tibiofemoral flexion angle the adjustment in the body's center of gravity resulted in an altered joint moment and a peak stress between 55% and 75% of the gait cycle at a time where stresses alter significantly. Then, as the gait cycle proceeded into next one and the center of gravity was successfully transferred, the tibiofemoral flexion angle gradually decreased, thereby resulting in a reduced patellar equivalent stress.

## 4. Conclusions

In this study, the kinematics and equivalent stress of two knee models (patellar retaining and patellar replacement) were compared during the gait cycle. Results showed the following: (1) patellar replacement has little influence on tibiofemoral kinematics and mechanical properties, although it produced a slightly increased tibial tray stress; (2) patellar replacement significantly reduced the kinematics and mechanical performances in the patellofemoral joint during the gait cycle; patellar tilting, internal rotation, and anterior-posterior translation were all significantly increased compared to the patellar retaining method; meanwhile, patellar replacement altered the stress distribution in the patellofemoral joint, resulting in an increased high-stress area; finally (3) compared to patellar retaining TKA, patellar replacement has less influence both on kinematic variations in the tibiofemoral and patellofemoral joints and on the variations in the joint peak equivalent stress during the gait cycle. In conclusion, TKA was shown to preserve human knee tissue following surgery. Moreover, the results here indicate that the patellar retaining method has more beneficial long-term effects compared to patellar replacement.

But this paper only analyzes the kinematics and mechanics with simplified knee models; this is only part of properties of total knee after TKA surgery. Furthermore, if the result is verified by some experiment, the paper will be more meaningful.

## Figures and Tables

**Table 1:** The stiffness coefficients of MCL and LCL.

Ligament	*K*_1_	*K*_2_
MCL anterior bundle	10	91.25
MCL deep bundle	5	21.07
MCL oblique bundle	5	27.86
LCL	10	72.11

**Table 2:** Knee tibial tray mechanical properties during the gait cycle.

	Gait cycle (patellar retaining)				Gait cycle (patellar replacement)			
	Heel strike	Single limb stance 1	Single limb stance 2	Toe off	Heel strike	Single limb stance 1	Single limb stance 2	Toe off
Flexion angle	4°	16°	6°	8°	4°	16°	6°	8°
Axial force (N)	1531	2482	1636	2434	1531	2482	1636	2434
Peak contact stress (MPa)	13.36	16.35	19.51	24.63	14.39	19.43	22.37	28.07
Stress increment					7.71%	18.84%	14.66%,	13.97%
High stress area	Medial	Medial	Medial and lateral	Medial and lateral	Medial	Medial	Medial and lateral	Medial and lateral
